# Changes in patient-reported chief complaints with orthognathic surgery: a prospective cohort study

**DOI:** 10.1186/s40510-025-00572-4

**Published:** 2025-07-22

**Authors:** Lennart Stadtmann, Moritz Kanemeier, Thomas Stamm, Claudius Middelberg, Carolien A. J. Bauer, Johannes Kleinheinz, Jonas Q. Schmid

**Affiliations:** 1 University of Münster, Münster, Germany; 2 Heidelberg University, Heidelberg, Germany

**Keywords:** Patient-reported outcome, Chief complaint, Orthodontics, Orthognathic surgery, Malocclusion, Aesthetics, Function, Pain, Temporomandibular disorder, TMD

## Abstract

**Objective:**

There is a lack of studies on patient-reported outcomes in orthodontics. The aim of this study was to evaluate the changes in patient-reported chief complaints during orthognathic surgery treatment.

**Materials and methods:**

Patients undergoing orthognathic surgery at the University Hospital Münster between 2019 and 2023 were eligible for inclusion in this prospective cohort study. Patient-reported chief complaints were recorded on visual analogue scale (VAS) forms before treatment ($\text {T}_{0}$), and reevaluated after presurgical orthodontic treatment ($\text {T}_{1}$), and 6–9 months after surgery ($\text {T}_{2}$). Chief complaints were grouped into three main categories (pain, function, aesthetics) and ten subcategories, and their intensity was quantified over time.

**Results:**

A total of 217 out of 386 recruited patients (56%) completed all study assessments and were included in the final analysis (female/male = 126/91, median age 24.1 years). Dental function, facial aesthetics, and dental aesthetics were the most frequently reported complaints at $\text {T}_{0}$. At $\text {T}_{1}$, there was a statistically significant improvement in dental aesthetics. There was a statistically significant reduction in the intensity of each of the 10 subcategories from $\text {T}_{0}$ to $\text {T}_{2}$.

**Conclusion:**

Orthognathic surgery patients most frequently report dental function, facial aesthetics, and dental aesthetics as their chief complaints, and these complaints were improved significantly after treatment.

**Clinical relevance:**

The improvement in patient-reported chief complaints can be used to inform patients prior to treatment.

**Supplementary Information:**

The online version contains supplementary material available at 10.1186/s40510-025-00572-4.

## Introduction

Malocclusion is a condition with a misalignment of the teeth and/or incorrect relationship between the jaws [1]. Its global prevalence in the permanent dentition is high [2] and it can lead to problems with masticatory performance [3], swallowing [4], speech [5], and reduced oral health related quality of life (OHRQoL) [6]. Orthognathic surgery is indicated if the malocclusion is too severe to be corrected by orthodontic treatment alone [7].

Orthognathic surgery patients report a wide range of chief complaints [8, 9] that vary considerably in severity between individuals [10]. Malocclusion-related complaints can be broadly divided into three categories: aesthetics [8], function [9], and pain [11].

Aesthetic concerns are often a major motivator for patients seeking orthognathic surgery treatment [8, 12–14]. Studies have shown that orthognathic surgery treatment can improve quality of life (QoL) in general [15–17].

Temporomandibular disorders (TMD) and pain are frequently found in patients with malocclusion [18]. The prevalence of TMD in orthognathic surgery patients before the operation is 32.5%, which is significantly higher than in a control population, but after treatment the prevalence of TMD does not differ from that of a control population [19]. The reason for this result remains unclear, as the association between malocclusion and TMD is questionable [20–24]. Orthognathic surgery appears to have little or no harmful effect on the temporomandibular joint [25], but the literature is inconclusive [19].

While orthodontic research has historically focused on clinician-centred outcomes such as cephalometric measurements [26–28] and occlusal indices [29, 30], there is a great need for studies that include patient perceptions of outcomes [14] and self-perceived aesthetics [31]. To date, there is a lack of evidence to determine which of the patient-reported complaints can be effectively addressed by orthognathic surgery. The present study adds to the literature in this regard.

The aim of this study was to categorise patient-reported chief complaints in orthognathic surgery patients and to evaluate changes during treatment. The null hypothesis was tested that orthognathic surgery does not lead to a significant change in patient-reported chief complaints over the course of orthognathic surgery treatment.

## Methods

This prospective cohort study received approval from the Ethics Commission of the Medical Faculty of the University of Münster, Germany (2019-334-f-S) and was reported according to the STROBE guidelines [32].

Patients consecutively seeking orthognathic surgery at the University Hospital of Münster between 2019 and 2023 were eligible for inclusion in this study. Patients who presented at the special consultation for orthognathic surgery at the Department of Cranio-Maxillofacial Surgery and the Department of Orthodontics were invited to participate.

The inclusion criteria were: (1) Adult patients, (2) clinical indication for orthognathic surgery, (3) treatment with fixed appliances, (4) treatment with LeFort I or bilateral sagittal split osteotomies (BSSO) as either single-jaw or two-jaw procedures, (5) informed consent to participate in this study.

The exclusion criteria were: (1) Treatment with any form of segmental osteotomy, as well as distraction osteogenesis procedures, LeFort II or III surgeries and surgery only approaches, (2) patients with craniofacial syndromes and clefts, (3) treatment with removable appliances, (4) no consent to participate, (5) patients with incomplete data were subsequently excluded.

All cases included in the study were planned with the Digital Münster Model Surgery (DMMS) system [33, 34] in the Department of Orthodontics and underwent orthognathic surgery in the Department of Cranio-Maxillofacial Surgery. The pre- and postoperative orthodontic treatment was provided either by the Department of Orthodontics or an external referring clinician.

### Sample size calculation

A sample size calculation using the pwr package [35] for R based on $\alpha = 0.05$ and a power of $1 - \beta = 0.90$ was performed. A pilot study was conducted to estimate the standard deviation of VAS scores, which was found to be 4.0. Assuming a clinically meaningful difference in VAS scores of 1.0, an effect size (Cohen’s d) of 0.25 was calculated. These values suggested that a minimum of 170 participants was required. Considering a potential dropout rate of 20%, the target sample size was adjusted to 204 participants.

### Data collection

A first rating was done at the initial pre-treatment consultation ($\text {T}_{0}$). Patients were asked to write down all chief complaints prior to any intervention. Printed blank visual analogue scale (VAS) forms were used to allow the patient to rate the severity of each complaint. This approach ensured that the primary complaints articulated by patients were captured in their original form and quantified at the time of reporting.

In order to accurately monitor the change in these chief complaints over the course of treatment, the severity rating of the same complaints was repeated at two additional time points. The second assessment ($\text {T}_{1}$) was made after the presurgical orthodontic treatment and just before surgery, when the patients attended the special consultation to receive an appointment for the operation. At this time, decompensation of the malocclusion can be assumed to be at its maximum.

A final rating ($\text {T}_{2}$) was done in the same way 6–9 months after surgery, when the patients attended the special consultation and were given an appointment for the removal of the osteosynthesis plates. At this stage, all active orthodontic appliances had already been removed, and no participants were undergoing further orthodontic treatment.

All questionnaires were digitised directly after the survey and subsequently analysed using NIH ImageJ software [36] (https://imagej.net/downloads). The VAS were evaluated on a range from 0 to 10, with 0 indicating no intensity and 10 indicating the maximum intensity. Each VAS could be distinctly allocated to a patient and a survey time in a digital data table by utilising an identifier and a date.

### Data categorisation

To facilitate the analysis of the free text chief complaints, a systematic categorisation process was employed. For this purpose, three main categories (pain, function and aesthetic) were defined and subdivided according to the anatomical region, resulting in the following ten categories: pain dental, pain facial, pain head, pain cervical spine, pain other, function dental, function facial, function other, aesthetic dental, aesthetic facial. The region"other"refers to any reported region caudal to the cervical spine.

All patient-reported chief complaints were then categorised by an orthodontic specialist into one of the ten predefined categories.

### Statistical analysis

The change in intensity over the three time points was analysed using linear mixed-effects models to account for repeated measurements within patients and multiple complaints per patient. A two-way interaction between time and complaint category was included to evaluate how changes in complaint intensity varied across categories. Fixed effects in the model included age, gender, and total treatment time, while random intercepts were specified for complaints nested within patients to model the hierarchical data structure. Model assumptions were assessed by examining residual plots, including Q–Q plots for normality and residuals versus fitted values for homoscedasticity. Slight deviations at the tails were noted in the Q–Q plots but were judged to be acceptable. The residuals versus fitted values plot showed diagonal boundaries reflecting the natural limits of the 0–10 VAS scale.

Estimated marginal means (EMMs) were derived from the fitted models to summarise complaint intensity at each time point, and model-based contrasts between time points were used to assess the significance of changes over time. In order to also assess the changes in complaint intensity separately by complaint type only (averaging across regions), an additional model was specified with the interaction restricted to time and complaint type, and region as a covariate.

In order to statistically assess the reliability of the categorisation of the complaints, a second rater (specialist in maxillofacial surgery) categorised 10% of randomly selected complaints. An inter-rater Cohen’s kappa test was performed to determine the level of agreement, interpreted according to Landis and Koch [37].

All statistical analyses were conducted using R (version 4.4.2) with the packages lme4, emmeans, and irr [38–41], with a significance level set at 0.05.

## Results

**Fig. 1:**
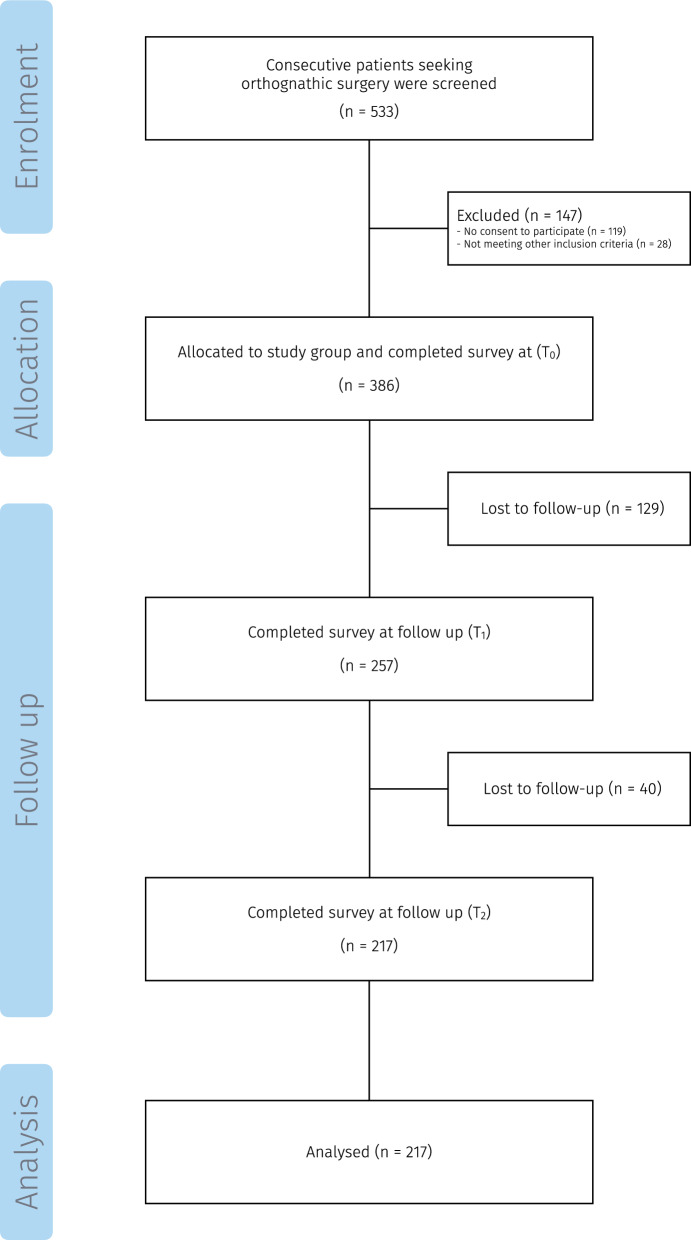
Flowchart of participants

In total, 386 patients were enrolled at $\text {T}_{0}$, of whom 217 (56%) completed all study assessments at each time point and were included in the final analysis (female/male = 126/91, median age 24.1 years). A flowchart of participants is shown in Fig. 1 and the baseline characteristics for included patients and those lost to follow up are presented in Table 1 and Table 2 respectively. The median age of the participants was 24.1 years (IQR: 19.9–30.9) and the median treatment time ($\text {T}_{0}$–$\text {T}_{2}$) was 3.0 years (IQR: 2.3–3.8).

**Table 1:** Baseline characteristics (number of patients by gender, Angle class, and surgical procedure; Median and IQR of age, treatment time, and sagittal correction)

	Female	Male	Total
n	126	91	217
Age	24.2 (19.8–31.5)	23.7 (20.0–30.3)	24.1 (19.9–30.9)
Treatment time	3.0 (2.3–3.8)	3.1 (2.2–3.8)	3.0 (2.3–3.8)
Angle class			
I	24 (19.0%)	9 (9.9%)	33 (15.2%)
II	62 (49.2%)	28 (30.8%)	90 (41.5%)
III	40 (31.7%)	54 (58.2%)	94 (43.3%)
Surgical procedure			
Le Fort I	1 (0.8%)	2 (2.2%)	3 (1.4%)
BSSO	41 (32.5%)	20 (22.0%)	61 (28.1%)
Le Fort I + BSSO	84 (66.7%)	69 (75.8%)	153 (70.5%)
Sagittal correction			
Angle class I	0.0 (0.0–0.1)	0.0 (0.0–0.0)	0.0 (0.0–0.0)
Angle class II	4.7 (3.7–6.0)	5.8 (3.6–6.3)	5.1 (3.6–6.1)
Angle class III	4.5 (3.5–7.2)	6.0 (3.7–8.2)	5.2 (3.5–8.1)

**Table 2:** Baseline characteristics of patients that were lost to follow up (number of patients by gender, Angle class, and surgical procedure; Median and IQR of age, and sagittal correction)

Lost to follow-up between	$T_0 \rightarrow T_1$	$T_1 \rightarrow T_2$
n	129	40
female	77 (59.7%)	18 (45.0%)
male	52 (40.3%)	22 (55.0%)
Age	24.1 (19.5–31.0)	23.9 (22.1–30.8)
Angle class		
I	9 (7.0%)	7 (17.5%)
II	51 (39.5%)	23 (57.5%)
III	69 (53.4%)	10 (25.0%)
Surgical procedure		
Le Fort I	–	0 (0.0%)
BSSO	–	23 (57.5%)
Le Fort I + BSSO	–	17 (42.5%)
Sagittal correction		
Angle class I	–	0.0 (−0.4 to 0.5)
Angle class II	–	3.9 (3.5–5.0)
Angle class III	–	6.1 (5.3–8.1)

**Fig. 2:**
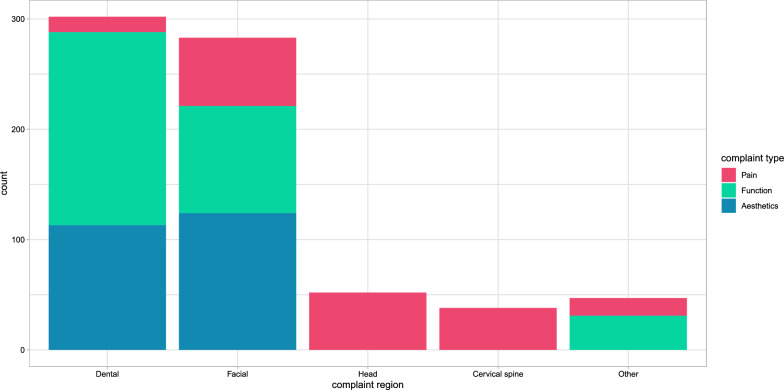
Patient-reported complaints categorised by type and region

The level of agreement for the categorisation of chief complaints was almost perfect ($\kappa$ = 0.84). 722 chief complaints were recorded at $\text {T}_{0}$ and re-assessed at $\text {T}_{1}$ and $\text {T}_{2}$, resulting in a total of 2166 ratings. The distribution of complaints by category and region is shown in Fig. 2. A total of 182 pain complaints, 303 functional complaints and 237 aesthetic complaints were recorded. 302 of the complaints related to the dental region, 283 to the facial region, 52 to the head, 38 to the cervical spine and 47 to other regions.

Table 3 shows the estimated VAS scores for complaint intensity of the three main categories (pain, function, aesthetics) at $\text {T}_{0}$–$\text {T}_{2}$. The corresponding model estimates are detailed in Online Table A1. Overall, there was a statistically significant reduction in complaint intensity across all categories over the observed time period. Pre-treatment ($\text {T}_{0}$), the intensity of complaints was comparable between categories, with aesthetics having the highest intensity. With presurgical orthodontic treatment ($\text {T}_{0}$–$\text {T}_{1}$), there was a significant improvement for pain and aesthetics, but not for function. After orthognathic surgery ($\text {T}_{0}$–$\text {T}_{2}$), there was a significant improvement in all three categories, with the greatest improvement in aesthetics.

**Table 3:** Estimated marginal means (EMMs) and 95% confidence intervals (CIs) for complaint intensity over time, based on a mixed-effects model with a two-way interaction between time and complaint type

Complaint Type	Intensity (EMM [95% CI])						Estimated Differences (Cohen’s d [95% CI])					
	$T_0$		$T_1$		$T_2$		$T_0 \rightarrow T_1$		$T_1 \rightarrow T_2$		$T_0 \rightarrow T_2$	
Pain	6.2	[5.7, 6.6]	5.3	[4.9, 5.7]	2.1	[1.7, 2.5]	$$0.3^{**}$$	[0.1, 0.5]	$$1.3^{***}$$	[1.1, 1.5]	$$1.6^{***}$$	[1.4, 1.9]
Function	5.8	[5.4, 6.2]	5.7	[5.3, 6.1]	1.8	[1.4, 2.2]	0.0	[– 0.1, 0.2]	$$1.6^{***}$$	[1.4, 1.7]	$$1.6^{***}$$	[1.4, 1.8]
Aesthetics	6.9	[6.5, 7.3]	5.4	[5.0, 5.8]	1.1	[0.7, 1.5]	$$0.6^{***}$$	[0.4, 0.8]	$$1.7^{***}$$	[1.5, 1.9]	$$2.3^{***}$$	[2.1, 2.5]

Estimated differences between time points are reported as standardized effect sizes (Cohen’s d) with 95% CIs. The model was adjusted for age, gender, total treatment time, and complaint region, with random intercepts for complaints nested within patients

$$^{*}p<0.05$$; $$^{**}p<0.01$$; $$^{***}p<0.001$$

**Fig. 3:**
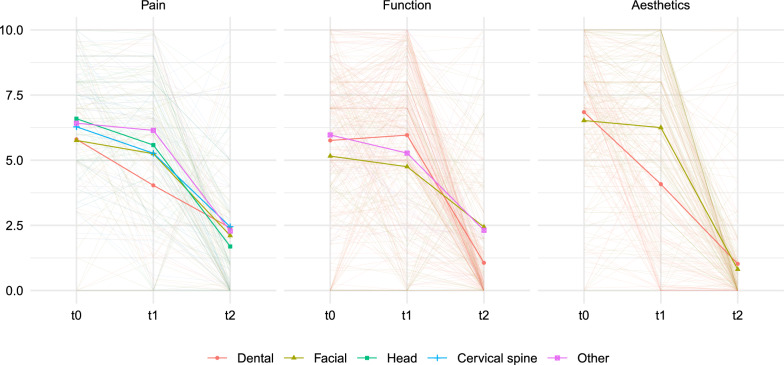
Spaghetti plot showing individual patient complaint intensity trajectories over time, faceted by complaint type and colored by complaint region. Semi-transparent lines represent raw individual data, while overlaid points indicate estimated marginal means (EMMs) from the mixed-effects model adjusted for age, gender, and total treatment time

Table 4 shows the estimated VAS scores for complaint intensity over all ten subcategories at $\text {T}_{0}$–$\text {T}_{2}$. The corresponding model estimates are detailed in Online Table A2. Overall, there was a statistically significant reduction in complaint intensity for all types and regions over the course of orthognathic surgery treatment ($\text {T}_{0}$–$\text {T}_{2}$),. These trends are visualized in Fig. 3, which displays individual trajectories and estimated means across timepoints. The greatest reduction was found for dental aesthetics, followed by facial aesthetics.

At baseline ($\text {T}_{0}$), VAS scores were high in all categories. The highest intensity was found for dental aesthetics, and the lowest intensity was found for facial function. For aesthetics, median VAS scores were slightly higher for dental aesthetics than for facial aesthetics. With presurgical orthodontic treatment ($\text {T}_{0}$–$\text {T}_{1}$), there was a statistically significant reduction in complaint intensity for dental aesthetics.

At $\text {T}_{1}$, the highest intensity was found for facial aesthetics, and the lowest intensity was registered for dental pain. Between $\text {T}_{1}$–$\text {T}_{2}$, there was a statistically significant improvement in all categories and regions except for dental pain. The highest intensity at $\text {T}_{2}$ was found for dental pain, cervical spine pain and facial function, and complaint intensity was lowest now for facial aesthetics.

**Table 4:** Estimated marginal means (EMMs) and 95% confidence intervals (CIs) for complaint intensity over time, based on a mixed-effects model with a two-way interaction between time and complaint category

Complaint Category		Intensity (EMM [95% CI])						Estimated Differences (Cohen’s d [95% CI])					
Type	Region	$T_0$		$T_1$		$T_2$		$T_0 \rightarrow T_1$		$T_1 \rightarrow T_2$		$T_0 \rightarrow T_2$	
Pain	Dental	5.8	[4.5, 7.1]	4.0	[2.7, 5.4]	2.4	[1.1, 3.7]	0.7	[0.0, 1.5]	0.7	[– 0.1, 1.4]	$$1.4^{***}$$	[0.7, 2.1]
	Facial	5.8	[5.1, 6.4]	5.3	[4.6, 5.9]	2.1	[1.5, 2.8]	0.2	[−0.1, 0.6]	$$1.3^{***}$$	[0.9, 1.7]	$$1.5^{***}$$	[1.2, 1.9]
	Head	6.6	[5.9, 7.3]	5.6	[4.9, 6.3]	1.7	[1.0, 2.4]	0.4	[0.0, 0.8]	$$1.6^{***}$$	[1.2, 2.0]	$$2.0^{***}$$	[1.6, 2.4]
	Cervical spine	6.3	[5.5, 7.1]	5.3	[4.4, 6.1]	2.4	[1.6, 3.3]	0.4	[0.0, 0.9]	$$1.2^{***}$$	[0.7, 1.6]	$$1.6^{***}$$	[1.1, 2.0]
	Other	6.4	[5.2, 7.7]	6.1	[4.9, 7.4]	2.3	[1.0, 3.5]	0.1	[– 0.6, 0.8]	$$1.6^{***}$$	[0.9, 2.3]	$$1.7^{***}$$	[1.0, 2.4]
Function	Dental	5.8	[5.4, 6.2]	6.0	[5.6, 6.4]	1.1	[0.7, 1.5]	−0.1	[– 0.3, 0.1]	$$2.0^{***}$$	[1.8, 2.2]	$$1.9^{***}$$	[1.7, 2.2]
	Facial	5.2	[4.6, 5.7]	4.8	[4.2, 5.3]	2.4	[1.9, 3.0]	0.2	[– 0.1, 0.4]	$$1.0^{***}$$	[0.7, 1.2]	$$1.1^{***}$$	[0.8, 1.4]
	Other	6.0	[5.1, 6.9]	5.3	[4.4, 6.2]	2.3	[1.4, 3.2]	0.3	[– 0.2, 0.8]	$$1.2^{***}$$	[0.7, 1.7]	$$1.5^{***}$$	[1.0, 2.0]
Aesthetics	Dental	6.8	[6.4, 7.3]	4.1	[3.6, 4.6]	1.0	[0.5, 1.5]	$$1.1^{***}$$	[0.9, 1.4]	$$1.3^{***}$$	[1.0, 1.5]	$$2.4^{***}$$	[2.1, 2.7]
	Facial	6.5	[6.1, 7.0]	6.3	[5.8, 6.7]	0.8	[0.3, 1.3]	0.1	[– 0.1, 0.4]	$$2.2^{***}$$	[2.0, 2.5]	$$2.4^{***}$$	[2.1, 2.6]

Estimated differences between time points are reported as standardized effect sizes (Cohen’s d) with 95% CIs. The model was adjusted for age, gender and total treatment time, with random intercepts for complaints nested within patients

$$^{*}p<0.05$$; $$^{**}p<0.01$$; $$^{***}p<0.001$$

## Discussion

The aim of this study was to evaluate changes in patient-reported chief complaints with orthognathic surgery treatment. The null hypothesis was rejected. There was a significant reduction in the intensity of complaints in all three categories (pain, function, aesthetics) over the study period. Regarding the ten subcategories, there was a statistically significant reduction in complaint intensity for all categories and regions over the course of orthognathic treatment. The greatest reductions were found for dental and facial aesthetics. Factors such as age and duration of treatment, did not significantly influence the outcomes, however gender had a significant influence on the change in complaint intensity, which could indicate possible gender-specific perception or reaction patterns during orthognathic surgery.

This finding is consistent with the available literature showing that orthognathic surgery treatment can improve QoL in general [15–17]. However, studies focus primarily on QoL and not on individual chief complaints [15]. To our best knowledge, there is a lack of evidence to determine which of the patient-reported complaints can be effectively addressed by orthognathic surgery. This is a clinically relevant topic which can be used to inform patients prior to orthognathic surgery. The present study adds to the literature in this regard. There is a great need in orthodontics for studies that include patient perceptions of outcomes [14] to assess what is most important to patients. According to the results of the present prospective study, patients can be informed that they can expect improvements in pain, function and aesthetics.

It is known that patients with malocclusion have individually different chief complaints, mainly aesthetic, functional or pain-related, and have different motivations for undergoing orthognathic surgery [10]. Therefore, when patients with malocclusion are asked about their chief complaints, it is reasonable to expect that each patient will identify multiple complaints with varying degrees of importance. To account for these individual complaints, this study did not use pre-defined categories or questionnaires, but instead asked patients to describe their complaints freely. These freely formulated complaints could still be analysed by subsequent validated categorisation. It is reasonable to expect a specialist to be able to categorise individual descriptions of complaints in a comparable way, since the result of Cohen’s kappa test with a score above 0.8 showed almost perfect agreement between two independent raters [37]. This study design has the advantage of measuring patient satisfaction as an endpoint rather than focusing on subject-specific parameters [14]. By analysing the improvement in complaints by category, a general statement can be made about each category, which can then be used by the clinician for individual patient counselling.

All three main categories showed similar levels of complaint intensity prior to treatment. There was no clear hierarchy, confirming the individual nature of malocclusion. Aesthetics tended to be rated higher than pain, closely followed by functional complaints. This is consistent with findings in the literature, where functional, aesthetic, social and psychological factors are most commonly mentioned as motivations for undergoing orthognathic surgery [13]. The results indicate that aesthetic complaints were on average reduced to a VAS score of about one and can therefore be almost completely corrected. This is consistent with previous studies reporting significant aesthetic improvements after orthognathic surgery [42]. The intensity of pain-related complaints did on average not improve below a VAS score of 2.1. The results indicate that orthognathic surgery alone is insufficient for a complete pain relief and patients should be informed accordingly. The results also indicate that dental function is easier to manage than facial or other function. This finding is also consistent with the current literature. Previous studies have shown that the improvement in patient perceived respiratory problems is independent of a change in upper airway volume [43]. Respiratory problems were categorised as other functional complaints in our study.

Presurgical orthodontic treatment leads to an inherent worsening of the malocclusion [44], as initially only the dental arches are aligned in relation to the individual jaws, in the sense of decompensation, in order to enable the subsequent correction of the skeletal malocclusion. This is reflected in the data, which showed no significant improvement in the intensity of functional complaints or in facial aesthetics following presurgical orthodontic treatment. In contrast, there was a significant improvement in dental aesthetics after presurgical orthodontic treatment, reflecting the therapeutic progress in the alignment of the dental arches.

With the surgical procedure ($\text {T}_{1}$–$\text {T}_{2}$), there were significant improvements in complaint intensity across most categories and regions. Specifically, function-related complaints improved significantly. The aesthetics category also improved substantially, particularly in the facial region, where the intensity of complaints was greatly reduced. This was expected, as orthognathic surgery can lead to a harmonisation of the profile [45, 46]. Overall, the surgical intervention had a positive impact on reducing various forms of complaints reported by patients.

Notably, there was a statistically significant reduction in pain over the observed time period. This is clinically relevant as the prevalence of pain and TMD in orthognathic surgery patients is high [19]. Our results confirm the results of Al-Moraissi et al. [19], who found that orthognathic surgery helped to reduce the prevalence of TMD symptoms, particularly pain. Orthognathic surgery appears to have little or no detrimental effect on the temporomandibular joint [25], but the literature is inconclusive [19]. However, the association between malocclusion and TMD is questionable [21, 47] and orthodontic treatment is considered to be TMD neutral [48]. As the aetiology of TMD has been described as multifactorial with a complex biopsychosocial aspect [21, 49], one possible explanation for the improvement observed in our study would be that orthognathic surgery treatment has a positive psychological impact on patients, with the implication that some form of therapy is being provided from the patient’s perspective. Taking into account the guidelines for the treatment of TMD, which discourage irreversible splinting [23], these results should be interpreted with caution. It seems difficult to predict the reduction in pain on an individual basis. Al-Moraissi et al. [19] also stated that orthognathic surgery is not a causal therapy for pain problems. It must be noted that the category pain had the highest post-treatment score in our study. However, this study supports the general concept of treating malocclusion-related complaints with orthognathic surgery. All of the complaint categories showed varying degrees of improvement from baseline.

### Strengths and limitations

The study has several strengths that make it a valuable addition to the literature. The prospective cohort design allowed data to be collected at predefined time points, provided a clear picture of treatment effects over the course of treatment, and reduced selection bias. Notably, the sample size of 217 patients was quite large, allowing the results to be more reliably generalised within the study context. Most studies on this topic have smaller sample sizes.

A major strength of the study is its patient-centred approach. It includes what is important to patients and focuses on patient-reported outcomes. A novel, highly individualised approach was chosen to record patients’ complaints qualitatively and quantitatively by combining free-text questionnaires with the established VAS [50]. Modern research in orthodontics should adopt a more patient-centred approach by including self-perceived aesthetics rather than reporting only clinician-centred metrics such as cephalometric values [14, 31]. This study adds to the literature in this area and offers insights that clinicians can use to better inform and counsel patients.

The comprehensive categorisation of complaints into pain, function, and aesthetics, with further sub-categorisation by region, allowed for concise analysis and ensured that specific patient concerns were addressed in detail. Although validated QoL tools provide summary measures of patient well-being, they have limitations, such as subjectivity, challenges in measurement and interpretation, issues with establishing clinical significance, and gaps in research and application to health policy [51]. Validated QoL tools do not always capture the predominance or change in very specific complaints that are important to individual patients. Our free-text methodology allows for direct analysis and quantification of these nuanced concerns. We cannot exclude the possibility that variability in the number and types of complaints reported per participant, as well as the use of single-item ratings, may have introduced bias or random error into our findings. Interdisciplinary collaboration between orthodontics and maxillofacial surgery ensured that data categorisation was both clinically relevant and methodologically sound. This was evident by the high level of agreement for the categorisation process ($\kappa$ = 0.84).

However, the study has certain limitations. The main limitation is the high drop-out rate. The fact that many patients could not be followed over three time points introduces a risk of bias, as there may be systematic differences between included and excluded participants, but we do not know the direction of this bias. We explain the high dropout rate with the COVID-19 pandemic, which caused many difficulties with appointments. Many appointments at the special consultation for orthognathic surgery were cancelled by the patients or had to be cancelled by the university hospital due to limited capacity in the operating theatre, making follow-up very difficult. It should be noted that almost all patients underwent orthognathic surgery, even if they could not be followed up over three time points. In addition, a large number of patients refused to participate in the study, which we attribute to a high level of scepticism about medical research at the time.

The findings are based on assessments from a single university hospital in western Germany. The results may therefore not be fully generalisable to other areas or healthcare settings with different patient populations and surgical protocols.

Additionally, the study lacks detailed analysis of factors such as psychological well-being and socio-economic status, which limits the ability to relate the results to specific patient subgroups. Future studies should aim to include these variables to allow for a more comprehensive understanding of patient experiences in orthognathic surgery. Variability in treatment approaches due to the involvement of multiple orthodontic practitioners could also introduce a risk of bias. The follow-up period of 6-9 months post-surgery may not fully capture the long-term patient-reported outcomes, leaving open the question of whether these benefits will persist or whether symptoms might recur with time. Further studies with long-term follow-up are necessary to answer these open questions. Although gender was identified as a significant influencing factor in the modelling, the study was not primarily designed to systematically investigate gender-specific differences. Therefore, future studies should specifically analyse gender-specific effects. Lastly, standardised questionnaires could have been used to improve the comparability and generalisability of the study. We did not specifically assess the reliability or test-retest consistency of participant complaint ratings, which may have introduced additional measurement variability.

## Conclusion

Orthognathic surgery patients most frequently report dental function, facial aesthetics, and dental aesthetics as their chief complaints, and these complaints were improved significantly after treatment. The improvement in patient-reported chief complaints can be used to inform patients prior to treatment.

## Supplementary Information

Supplementary file

## Data Availability

The data presented in this study are available on reasonable request from the corresponding author.
