# Supplementary material for: Changes in patient-reported chief complaints with orthognathic surgery: a prospective cohort study
Source: Prog Orthod. 2025 Jul 22;26:27. doi: 10.1186/s40510-025-00572-4 (PMC12283525; doi:10.1186/s40510-025-00572-4)
Supplement: Supplementary file 1 — Supplementary file [file 40510_2025_572_MOESM1_ESM.pdf]

Supplementary Data

Table A1: Fixed effect estimates from the linear mixed-effects model assessing complaint intensity over time by complaint type. The model includes an interaction between time and complaint type, and adjusts for age, total treatment time, gender, and complaint region. Random intercepts were specified for complaints nested within individuals. Estimates are presented with standard errors (SE), 95% confidence intervals (CI), and Wald p-values.

Parameter	Estimate	SE	95% CI		p
(Intercept)	0.29	0.38	-0.46	1.04	0.451
Age	0.01	0.01	-0.01	0.03	0.241
Total treatment time	-0.05	0.07	-0.19	0.09	0.471
Gender					
female	0.63	0.18	0.27	0.99	<0.001
male	0.0[a]	-	-	-	-
Complaint region					
Dental	0.0[a]	-	-	-	-
Facial	0.19	0.14	-0.08	0.46	0.164
Head	0.39	0.29	-0.18	0.97	0.180
Cervical spine	0.44	0.32	-0.19	1.07	0.170
Other	0.52	0.26	0.00	1.04	0.051
Time × Complaint type					
T_0 × Pain	5.04	0.29	4.48	5.60	<0.001
T_1 × Pain	4.21	0.29	3.65	4.77	<0.001
T_2 × Pain	0.97	0.29	0.41	1.53	<0.001
T_0 × Function	4.67	0.23	4.23	5.11	<0.001
T_1 × Function	4.58	0.23	4.14	5.03	<0.001
T_2 × Function	0.71	0.23	0.27	1.15	0.002
T_0 × Aesthetics	5.76	0.23	5.32	6.21	<0.001
T_1 × Aesthetics	4.30	0.23	3.86	4.75	<0.001
T_2 × Aesthetics	0.0[a]	-	-	-	-

[a]Reference category (coefficient fixed to 0).

Table A2: Fixed effect estimates from the linear mixed-effects model assessing complaint intensity over time by complaint category. The model includes an interaction between time and complaint category, and adjusts for age, total treatment time, and gender. Random intercepts were specified for complaints nested within individuals. Estimates are presented with standard errors (SE), 95% confidence intervals (CI), and Wald p-values.

Parameter	Estimate	SE	95% CI		p
(Intercept)	0.28	0.41	-0.51	1.08	0.486
Age	0.01	0.01	-0.01	0.03	0.236
Total treatment time	-0.05	0.07	-0.19	0.10	0.519
Gender					
female	0.62	0.18	0.26	0.97	<0.001
male	0.0[a]	-	-	-	-
Time × Complaint category					
T_0 × Dental Pain	4.99	0.71	3.59	6.39	<0.001
T_1 × Dental Pain	3.22	0.71	1.82	4.62	<0.001
T_2 × Dental Pain	1.60	0.71	0.20	3.00	0.025
T_0 × Facial Pain	4.95	0.39	4.17	5.72	<0.001
T_1 × Facial Pain	4.44	0.39	3.67	5.21	<0.001
T_2 × Facial Pain	1.30	0.39	0.52	2.07	0.001
T_0 × Head Pain	5.78	0.42	4.96	6.60	<0.001
T_1 × Head Pain	4.77	0.42	3.95	5.59	<0.001
T_2 × Head Pain	0.88	0.42	0.06	1.69	0.035
T_0 × Cervical spine Pain	5.47	0.47	4.55	6.39	<0.001
T_1 × Cervical spine Pain	4.45	0.47	3.53	5.37	<0.001
T_2 × Cervical spine Pain	1.63	0.47	0.71	2.55	<0.001
T_0 × Other Pain	5.61	0.68	4.28	6.94	<0.001
T_1 × Other Pain	5.33	0.68	4.01	6.66	<0.001
T_2 × Other Pain	1.47	0.68	0.14	2.80	0.030
T_0 × Dental Function	4.95	0.30	4.37	5.53	<0.001
T_1 × Dental Function	5.15	0.30	4.57	5.73	<0.001
T_2 × Dental Function	0.25	0.30	-0.33	0.83	0.397
T_0 × Facial Function	4.35	0.34	3.67	5.02	<0.001
T_1 × Facial Function	3.94	0.34	3.26	4.61	<0.001
T_2 × Facial Function	1.62	0.34	0.95	2.30	<0.001
T_0 × Other Function	5.16	0.52	4.15	6.17	<0.001
T_1 × Other Function	4.46	0.52	3.45	5.47	<0.001
T_2 × Other Function	1.50	0.52	0.49	2.51	0.004
T_0 × Dental Aesthetics	6.03	0.33	5.39	6.68	<0.001
T_1 × Dental Aesthetics	3.27	0.33	2.62	3.92	<0.001
T_2 × Dental Aesthetics	0.21	0.33	-0.44	0.86	0.523
T_0 × Facial Aesthetics	5.71	0.31	5.11	6.31	<0.001
T_1 × Facial Aesthetics	5.44	0.31	4.84	6.04	<0.001
T_2 × Facial Aesthetics	0.0[a]	-	-	-	-

[a]Reference category (coefficient fixed to 0).
